# dsRNA in immunometabolism

**DOI:** 10.18632/oncotarget.5100

**Published:** 2015-08-05

**Authors:** Takahisa Nakamura

**Affiliations:** Divisions of Endocrinology and Developmental Biology, Cincinnati Children's Hospital Medical Center, Cincinnati, Ohio, USA

**Keywords:** immunometabolism, obesity, double-stranded RNA, PKR, TRBP, Immunology and Microbiology Section, Immune response, Immunity

The worldwide prevalence of obesity has reached pandemic proportions, and with it have come other associated metabolic diseases, such as insulin resistance and type 2 diabetes (T2D). Intensive research has identified the frequent coexistence of obesity with a state of inflammation in metabolic tissues such as adipose tissue and liver [1]. Multiple inflammatory and stress responses are evoked in these metabolic tissues, leading to chronic, low grade, local inflammation that plays a central role in the disruption of systemic metabolic homeostasis during the pathogenesis of obesity. This atypical state engages immune response pathways, including recruitment of immune cells into metabolic tissues, activation of IB kinase (IKK) and c-Jun N-terminal kinase (JNK) pathways, and elevated production of an array of immune mediators that impact nutrient metabolism and insulin action [1]. However, the molecular basis for the induction of metabolic inflammation and the vast network of pathological responses remains to be elucidated.

In general, a majority of cellular double-stranded RNA (dsRNA)-binding proteins, such as dsRNAdependent protein kinase (PKR) and retinoic acid-inducible gene-I (RIG-I)-like receptor family, have been known to exclusively recognize exogenous dsRNA and induce inflammatory responses [2]. However, recent evidence has clearly shown that a variety of endogenous dsRNA exist and are involved in multiple cellular events including RNA silencing, which requires stringent regulation of dsRNA-to-dsRNA-binding protein interactions. As many dsRNA-binding proteins are intrinsically pro-inflammatory, regulatory disruption in endogenous dsRNA-protein networks may also cause inflammatory responses. We have hypothesized that such a disruption occurs in obesity, resulting in inflammation, insulin resistance, and metabolic dysfunction. This hypothesis has allowed us to identify PKR as a negative regulator of insulin action and glucose metabolism in obesity [3].

PKR, a pathogen-sensing protein, is activated by excess nutrients and its aberrant activity plays a key role in the induction of inflammatory responses, insulin resistance, and abnormal glucose metabolism in obesity [3]. In addition, exposure of cells and tissues to lipotoxicity leads to PKR activation, PKR-dependent activation of JNK, and phosphorylation of eukaryotic initiation factor 2α (eIF2α), a direct substrate of PKR [3]. Importantly, PKR and JNK activities as well as eIF2α phosphorylation are elevated in multiple tissues in obese humans [4], strongly suggesting that this role of PKR is conserved and relevant to human disease. Of note, we have recently reported that PKR interacts with specific endogenous dsRNA-like RNA including sets of small nucleolar RNA (snoRNA), which in turn activate PKR, in a PKR-dsRNA-binding domain dependent manner in lipotoxic conditions [5]. These data raise the possibility that metabolic stress may alter the quality and/or quantity of dsRNA and that these alterations are recognized by dsRNA-binding proteins, which then initiate inflammation and induce metabolic dysfunction.

More recently, we have identified TRBP, a trans-activation response (TAR) element-binding protein, as a physiologically-crucial component involved in obesity-induced PKR activation and metabolic dysfunction [6]. TRBP is known to be a unique dsRNA-binding protein that has at least two molecular functions; 1) enhancing microRNA (miRNA) maturation processes as a component of the RNA-induced silencing complex (RISC)-loading complex (RLC) and 2) regulating inflammatory responses by modifying PKR activity [7]. PKR forms a distinct complex with TRBP, and this complex preferentially assembles in states where PKR is activated, including the obese liver, polyinosinic-polycytidylic acid treatment, and palmitate exposure [6]. Importantly, TRBP is required for palmitate-induced PKR and JNK activation and eIF2α phosphorylation; these events are drastically diminished in the TRBP-deficient condition [6]. These data suggest that TRBP function is regulated by metabolic stress and the PKR-TRBP complex formation in response to nutrient signals; all of which are critical for key downstream events such as JNK activation. Consistent with these observations, acute inactivation of TRBP in the liver of *ob/ob* mice (a leptin-deficient obese mouse model) resulted in improved glucose tolerance, accompanied by significant reduction of JNK activity and eIF2α phosphorylation [6]. These findings indicate that TRBP is critical for regulation of these molecules and insulin action in metabolic stress.

Our study suggests that metabolic inflammation results, at least in part, from deranged dsRNA processing/signaling, which adversely regulates systemic glucose metabolism in obesity. Given that TRBP is a dsRNAbinding protein involved in multiple cellular events, TRBP may be the signaling node that senses metabolic stress levels through the recognition of dsRNA networks, and links to miRNA outputs and PKR-mediated inflammatory signaling networks under the stress conditions. Identifying the molecular basis by which changes of endogenous dsRNA dynamics trigger chronic inflammation would pave the way for developing novel therapeutic strategies for not only obesity-induced metabolic diseases but also other chronic inflammatory diseases.
